# Alternative LIM homeodomain splice variants are dynamically regulated at key developmental steps in vertebrates

**DOI:** 10.1002/dvdy.466

**Published:** 2022-03-18

**Authors:** Benjamin Joel Wheaton, Sara Lea Häggström, Mridula Muppavarapu, Luz María González‐Castrillón, Sara Ivy Wilson

**Affiliations:** ^1^ Department of Integrative Medical Biology Umeå University Umeå Sweden

**Keywords:** axon guidance, chick, Lhx2, Lhx9, mouse, neurodevelopment, neuron, spinal cord, splice, transcription factor, urogenital ridge

## Abstract

**Background:**

Alternative splicing provides a broad strategy to amplify the genome. Yet how alternative splicing influences neurodevelopment or indeed which variants are translated at developmental choice points remains poorly explored. Here we focused on a gene important for neurodevelopment, the Lim homeodomain transcription factor, Lhx9. Lhx9 has two noncanonical splice variants, Lhx9a and Lhx9b which compared with the canonical variant Lhx9c have a truncated homeodomain and an alternative C‐terminal sequence, suggesting that, if translated, these variants could differently impact on cellular function.

**Results:**

We created a unique antibody tool designed to selectively detect noncanonical Lhx9 variants (Lhx9ab) and used this to examine the protein expression dynamics in embryos. Lhx9ab variants were translated and dynamically expressed similarly between mouse and chicken at key developmental choice points in the spinal cord, limbs and urogenital ridge. Within the spinal cord, enrichment of *Lhx9c* vs Lhx9ab expression was observed during key migration and axonal projection choice points.

**Conclusions:**

These data support the notion that the expression dynamics between canonical and noncanonical Lhx9 variants could play an important role in spinal neuron maturation. More broadly, determining the temporal dynamics of alternative protein variants is a key entry point to understand how splicing influences developmental processes.

## Key Findings

- The LIM homeodomain transcription factor Lhx9, which is important for neural development has two non‐canonical splice variants, Lhx9a and Lhx9b which compared with the canonical variant Lhx9c have a truncated homeodomain and alternative C‐terminal sequence.
- An antibody was created which selectively recognises the non‐canonical Lhx9 spice variants (Lhx9ab) which was used to determine the spatiotemporal expression of non‐canonical Lhx9 variants during mouse and chicken embryogenesis.
- It was discovered that Lhx9ab non‐canonical variants were translated and detected at key developmental choice points in the spinal cord, urogenital ridge and limbs in a partly overlapping and partially independent manner to Lhx9 canonical transcripts.
- Within the spinal cord, Lhx9ab protein was detected in dI1 neurons at key choice divergence points for dI1c and dI1i neural projections suggesting that the expression dynamics between canonical and non‐canonical Lhx9 variants could play an important role at key development choice points within the spinal cord.
- More broadly, determining the temporal dynamics of alternative protein variants is a key entry point to understand how splicing influences developmental processes.

**Abbreviations**
dI1 neurons: dorsal interneuron 1 neurons
dI1c neurons: dorsal interneuron 1 commissural neurons
dI1i neurons: dorsal interneuron 1 ipsilateral neurons
LIM‐HD transcription factor: LIM homeodomain transcription factor
LMO protein: LIM domain only protein

## 1 INTRODUCTION

The nervous system is composed of billions of neurons that connect in a precise spatial relationship. The anatomical organization that underpins this is assembled during embryonic development and is instructed by molecular codes. While good progress has been made on understanding the genes that underlie this process, surprisingly little is known about how alternative splicing influences neural development, or in most cases even which variants of genes are expressed at key developmental choice points. This is critical since in mammals, an estimated 95% of genes undergo alternative splicing providing a broad strategy to amplify the functional genome.^1^ Given that different splice variants can result in proteins with different structures, activities and alternative functions, this feature could substantially impact the understanding of the molecular pathways underlying neural development and other biological processes. To examine this further, here we focused on a gene family known to be fundamental for neural and other aspects of development, Lim homeodomain (LIM‐HD) transcription factors.

**FIGURE 1:**
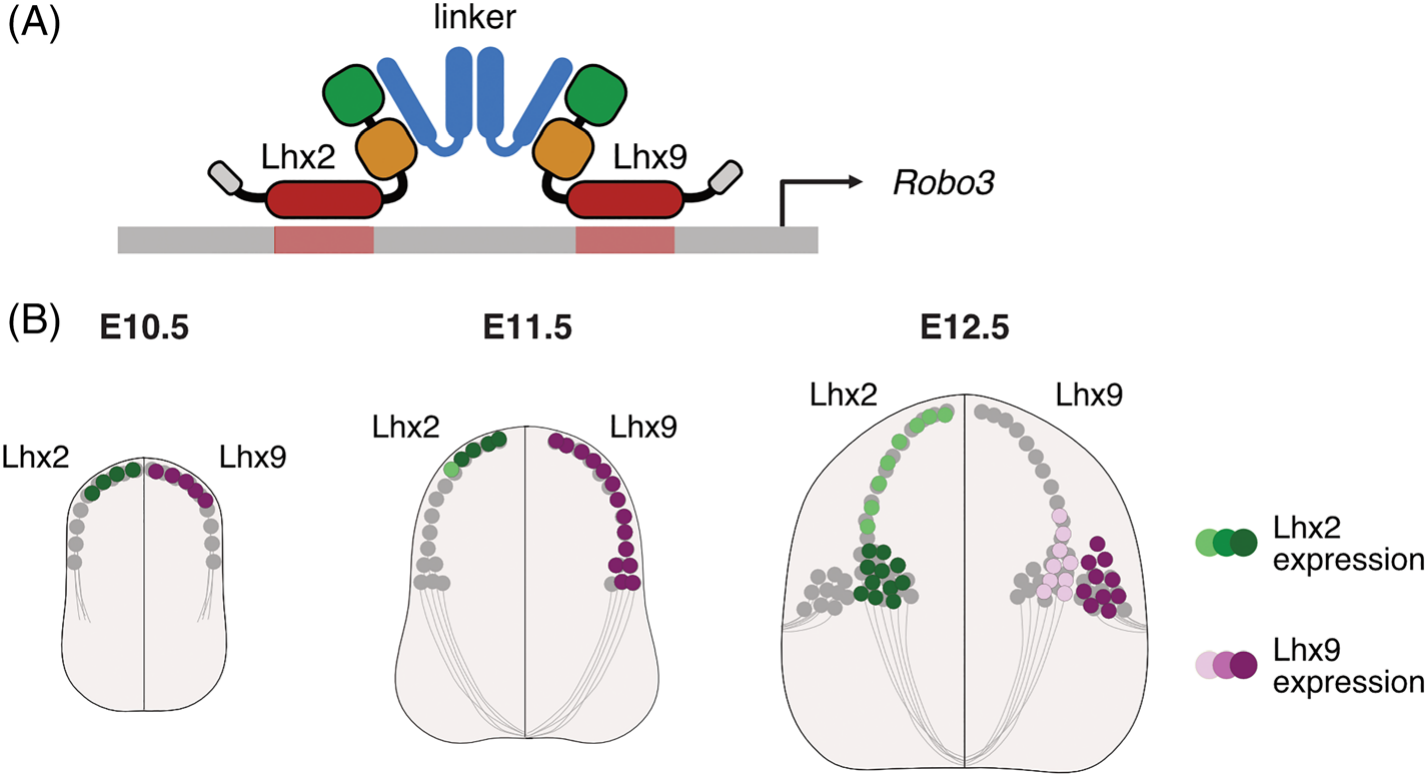
LIM‐HD transcription factors and spinal dI1 development. (A) Schematic representation of the LIM‐HD transcription factors Lhx2 and Lhx9 on the *Robo3* gene target site. In its simplest form, the transcription factors form a multimeric complex with linker proteins (blue) in order to function. Together this complex leads to transcriptional regulation. The LIM 1 (green) and LIM 2 (orange) domains and homeodomain (red) of Lhx2 and Lhx9c are indicated. (B) Schematic representation of the development of dI1 neurons, demonstrating the expression of Lhx2 (green) and Lhx9 (purple), key markers of dI1 neuron identity over developmental time

LIM‐HD transcription factors comprise a large family of proteins composed of six groups of related paralogues, Isl1/Isl2, Lhx1/Lhx5, Lhx2/Lhx9, Lhx3/Lhx4, Lhx6/Lhx8 (formerly called Lhx7), and Lmx1a/Lmx1b. These proteins are characterized by a stereotypical domain structure from the N‐C terminus composed of two zinc finger‐containing LIM domains important for allosteric interactions with other proteins, a homeodomain that binds to specific DNA sequences and an additional sequence C‐terminal to the homeodomain (Figure 1A). Classically LIM‐HD transcription factors act in multimeric complexes composed of at least two LIM‐HD transcription factors, joined together with a linker protein such as the Ldb family (Ldb1/CLIM2/NLI or Ldb2/CLIM1) and modulated by other proteins (Figure 1A). Within the multimeric complex, the homeodomain region of each LIM‐HD transcription factor binds to specific DNA sequences to elicit transcriptional regulation.^2^ Within the developing nervous system, LIM‐HD transcription factors are expressed in a neural subtype‐specific manner and act in combinatorial codes to elicit downstream neuron subtype‐specific developmental features such as neural guidance and positioning. The developing spinal cord where the precursor zone is divided along the dorsoventral axis with each respective precursor region giving rise to subpopulations of postmitotic neurons with specific LIM‐HD transcription factor codes provides a good illustration of this.^3^ For example, in developing in spinal motor neurons, the LIM‐HD paralogues Isl1 and Isl2 are central to motor neuron development whereas the paralogues Lhx9 and Lhx2 play a fundamental role in sculpting neuroanatomical features of spinal projection neurons (called dorsal interneuron 1 [dI1]).^4^, ^5^ dI1 neurons are derived from the developing dorsal spinal cord, and were originally defined by their selective expression of the LIM‐HD transcription factor paralogues Lhx2 and Lhx9.^6^, ^7^ During their development dI1 neurons differentiate into several anatomically distinct neural populations including dI1 commissural (dI1c) and dI1 ipsilateral (dI1i) neurons, which project axon either commissurally or ipsilaterally, respectively (Figure 1B). Interestingly while Lhx2 and Lhx9 are expressed in dI1 neuron when they are first generated, as dI1 neurons mature and anatomically diverge Lhx2 and Lhx9 become differentially expressed in different populations.^4^ Knocking out both *Lhx2* and *Lhx9* in mouse embryos results in a profound misguidance of the dI1c axons, a feature which is controlled by Lhx2 and Lhx9s transcriptional regulation of the axon guidance molecule *Robo3* (Figure 1A,B).^4^

**FIGURE 2:**
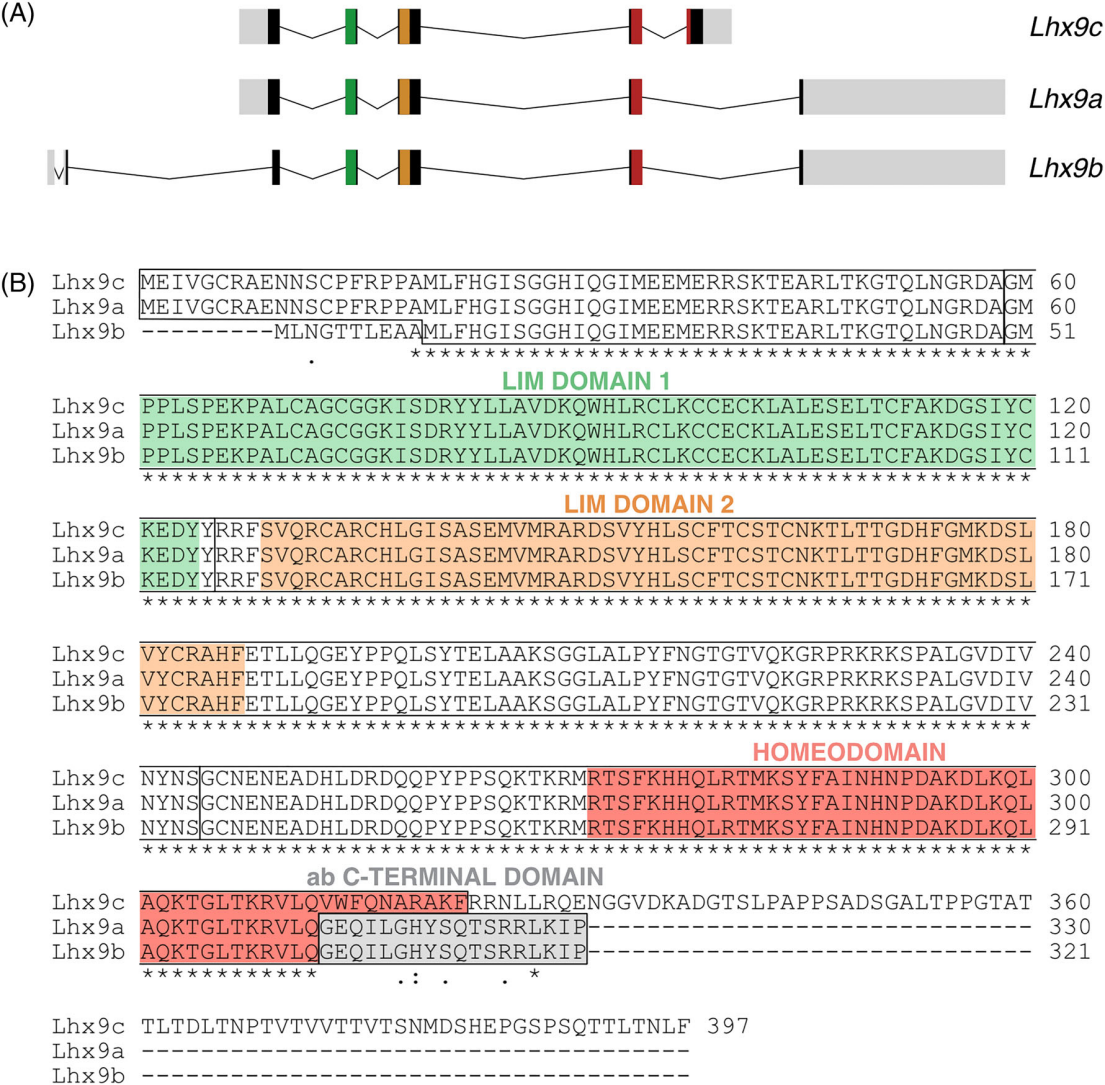
Lhx9 splice variants in mouse. (A) Schematic representation of the structure of *Lhx9* pre‐mRNA isoforms in mouse. *Lhx9c* (NCBI accession number NM_001042577.1), *Lhx9a* (NM_001025565.2) and *Lhx9b (*NM_0010714.3) are shown. Introns (black lines), exons (boxes), LIM 1 domain (green), LIM 2 domain (orange), homeodomain (red), undefined translated regions (black), and untranslated region (light gray) are indicated. (B) Protein sequence alignments of Lhx9 variants Lhx9c (NCBI accession number NP_001036042.1), Lhx9a (NP_001020736.1), and Lhx9b (NP_034844.1) in mouse. The LIM1 (green) and LIM2 (orange) domain are zinc finger domains which interact with proteins and the homeodomain (red) is the DNA binding region. The alternative C‐terminal domain of the Lhx9ab proteins is indicated (gray). Comparing all three variants (*) indicates positions which have a single, fully conserved residue, (:) indicates conservation between amino acids with similar properties and (.) indicates conservation between amino acids with weakly similar properties. Alignments were generated with CLUSTAL O (version 1.2.4)

Given the potency of LIM‐HD transcription factors, it is crucial to regulate their activity. One strategy could be by controlling the translation of splice variants, which give rise to structurally different variants of the gene. LIM‐HD transcription factors have a number of transcript variants and despite their potential importance in biological mechanisms, the differential expression and biological functions of transcription factor splice variation is poorly explored. Here we focused on the LIM‐HD transcription factor *Lhx9* where several transcript variants have been isolated in mouse including *Lhx9a*, *Lhx9b*, and *Lhx9c* (Figure 2A).^8^, ^9^, ^10^ Compared with the canonical Lhx9c secondary sequence which has a classical LIM‐HD domain composition, Lhx9a and Lhx9b protein variants have an alternative C‐terminal region that is missing the third helix of the homeodomain, thought to be critical for DNA binding and the region C‐terminal to the homeodomain is replaced by an alternative short sequence (Figure 2B).^10^, ^11^

Previous studies with noncanonical *Lhx9* gene variants have shown mRNA expression during heart, gonad, limb, and cortex development suggesting it could have a broad developmental function.^12^, ^13^, ^14^, ^15^, ^16^ However, given the tools available to date together with the knowledge that canonical and noncanonical *Lhx9* variants largely spatially overlap in these regions, it has not been possible to elucidate the protein distribution in a splice variant selective manner. Here we generated a unique antibody tool to monitor the dynamics of the noncanonical Lhx9 isoforms (Lhx9ab) and used it to determine spatial and temporal dynamics of Lhx9ab protein expression during mid‐gestation mouse and chicken development. We particularly focused on the spinal cord where Lhx9 plays an important role.^4^ We conclude that Lhx9ab is translated and expressed dynamically in the developing embryo coincident with major developmental events, suggesting a fundamental role for noncanonical Lhx9ab variants.

## 2 RESULTS

### 2.1 Noncanonical Lhx9 variants are present in a wide range of vertebrates including both mouse and chicken

**FIGURE 3:**
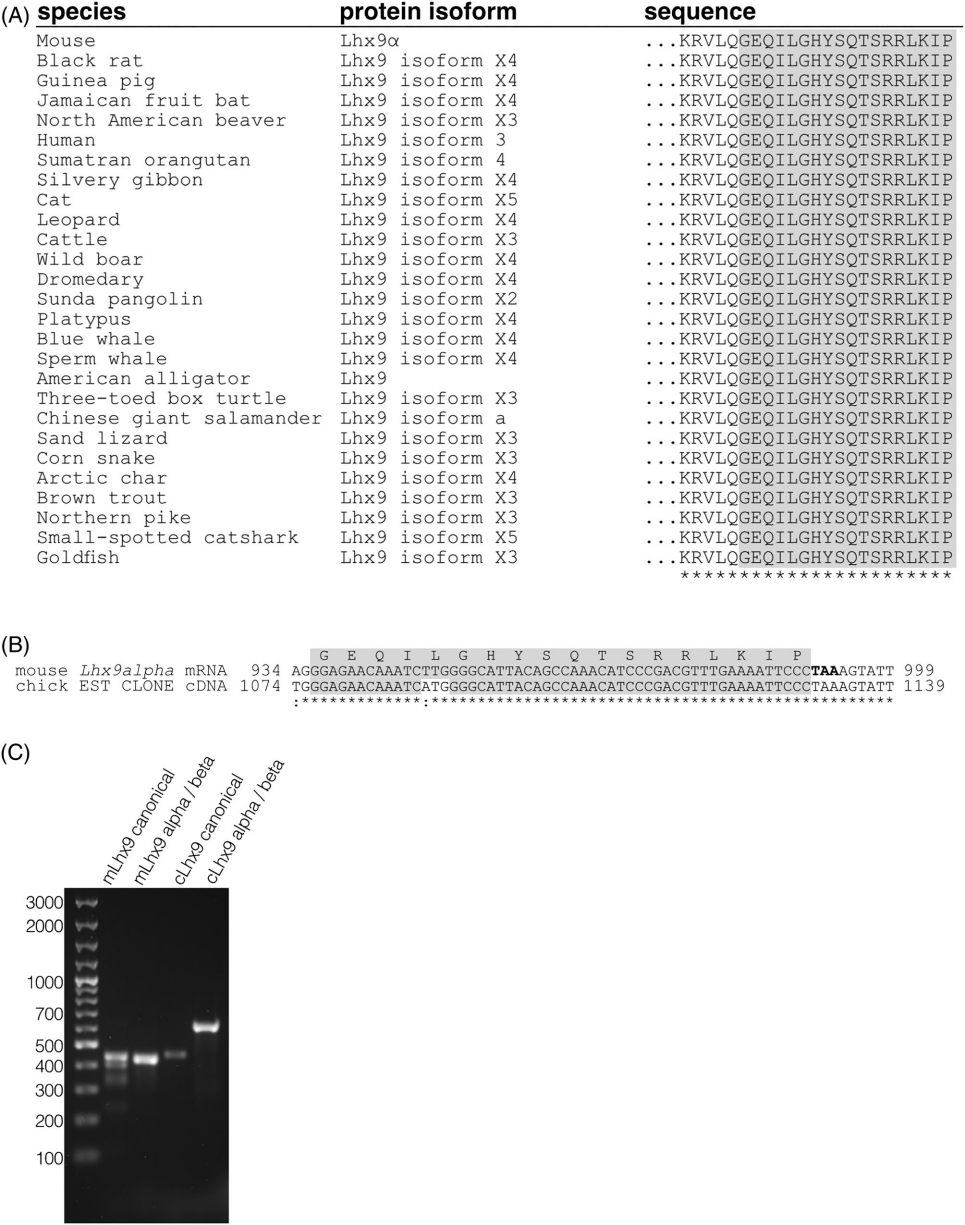
Lhx9 isoforms are expressed in different species. (A) A protein BLAST against the Lhx9a alternative C‐terminal domain “GEQILGHYSQTSRRLKIP” was performed. Different species where either verified or predicted proteins similar or identical to mouse Lhx9a alternative C‐terminal domain are shown. (B) A region of the alignment between the chick *EST* clone cDNA (NCBI accession number CR407573.1, clone ChEST96k16) and mouse *Lhx9a* mRNA (NM_001025565.2) is shown. The translated Lhx9 alternative C‐terminal domain is shown (gray) and the mouse *Lhx9a* stop codon is indicated in bold. (C) Mouse and chicken embryonic cDNA was analyzed for the presence of *Lhx9c* and *Lhx9ab* using PCR with oligonucleotides listed in Table 2. The gel image shows the following: molecular weight markers (lane 1), mouse *Lhx9* canonical (lane 2), mouse *Lhx9ab* (lane 3), chicken *Lhx9* canonical (lane 4) and chicken *Lhx9ab* (lane 5). The sizes of the bands in the marker lane are indicated. The gel image is representative of three biological replicates

In order to investigate canonical vs noncanonical *Lhx9* splice variants further, we first examined the presence of the noncanonical variants among different vertebrate species *in silico*. To do this the Lhx9ab specific sequence at the C‐terminal end of noncanonical variants “GEQILGHYSQTSRRLKIP” (referred to in this article as “Lhx9ab alternative C‐terminal sequence”) was used (Figure 2B). BLAST of the Lhx9ab alternative C‐terminal sequence against all species revealed predicted or experimentally identified Lhx9ab sequences for a wide range of vertebrate species including mammals, birds, fish and reptiles in most cases with 100% identity (Figure 3A). This included an unidentified chicken cDNA clone (ChEST96k16), derived from mRNA isolated from chicken embryo limbs, which contained a sequence that when translated in silico was highly similar the Lhx9ab alternative C‐terminal sequence, GEQIMGHYSQTSRRLKIP (Figure 3B). This provided evidence that an mRNA product corresponding to mouse *Lhx9ab* was expressed in chicken. We confirmed the presence of noncanonical *Lhx9ab* transcript experimentally in both mouse and chicken embryonic tissue using RT‐PCR (Figure 3C). Of note, consistent with a previous report, we did not detect this alternative end on any other LIM‐homeodomain homologue at the time of analysis.^10^ This suggested that this alternative C‐terminal domain was specific to the *Lhx9* gene. Taken together this provided evidence that the noncanonical *Lhx9* transcript variants were highly conserved among a wide range of amniote and anamniote species implying a fundamental role for this variant (Figures 2 and 3).

### 2.2 Lhx9ab protein is dynamically expressed in mouse and chicken embryos

The noncanonical splice variants Lhx9a and Lhx9b lack the third helix of the homeodomain present in the canonical Lhx9 splice variant (Lhx9c), suggesting distinct functions for canonical vs noncanonical variants during development. To examine Lhx9 splice variants further, two major developmental model systems were examined, mouse and chicken embryos. A small number of studies have shown *Lhx9ab* mRNA expression in the developing heart, limbs, gonads, and brain of mouse embryos whereas to our knowledge no information is available for chicken embryos. Importantly, the vast majority of studies of *Lhx9* expression and function use tools that will not distinguish between canonical and noncanonical splice variants and to date no study has determined the splice variant protein distributions using variant‐specific tools.^4^, ^9^, ^10^, ^12^, ^13^, ^15^, ^16^, ^17^, ^18^, ^19^ Therefore, we next sought to determine the distribution of Lhx9ab variants in developing embryos with a focus on the developing spinal cord.

**FIGURE 4:**
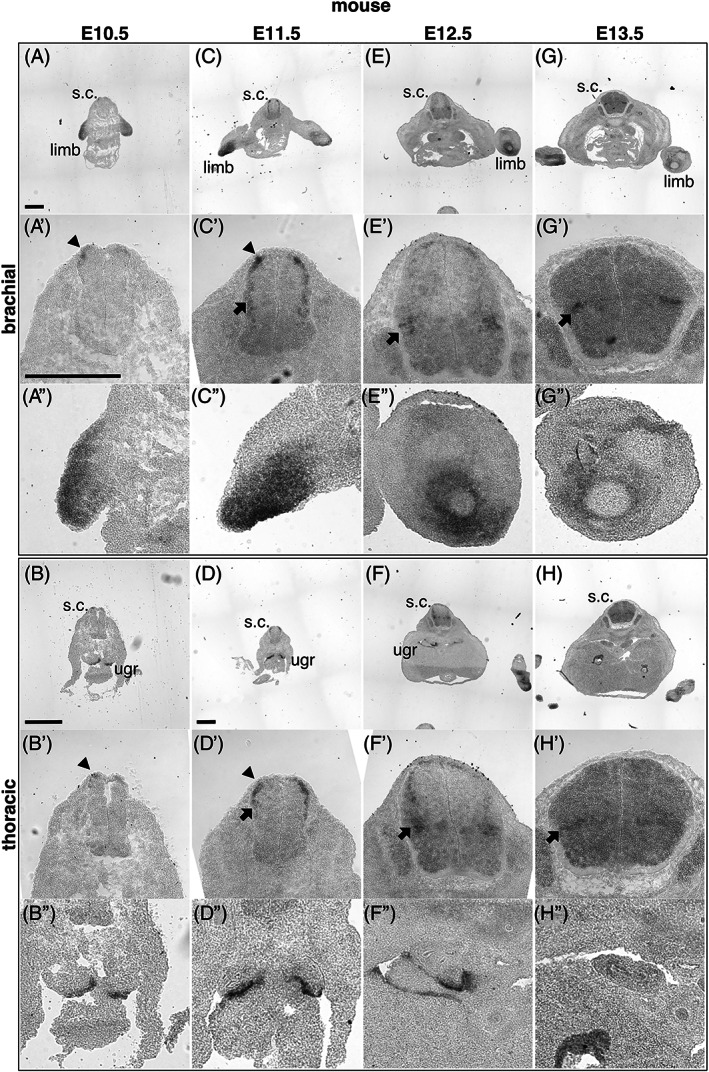
*Lhx9* is expressed in mid‐gestation mouse embryos. Brightfield images of brachial and thoracic transverse sections of mouse embryos at E10.5 (A, A′, A", B, B′, B″), E11.5 (C, C′, C″, D, D′, D″), E12.5 (E, E′, E″, F, F′, F″), and E13.5 (G, G′, G″, H, H′, H″) labeled by *in situ* hybridization with a probe against mouse *pan‐Lhx9*. The limb, spinal cord (s.c.), spinal cord dorsal mantel zone (black arrowhead), deep dorsal horn (black arrow), and urogenital ridge (ugr) are indicated. Higher magnification images are shown for the spinal cord (A′‐H′), the forelimb (A″, C″, E″, G″) and urogenital ridge (B″, D″, F″, H″). Representative images are shown. Three to five individual embryos were analyzed for each developmental age. Scale bars are 500 μm. The scale bars in A, A′, B, and D represent images in (A, C, E, G), (A′, A", B′, B″, C′, C″, D′, D″, E′, E″, F′, F″, G′, G″, H′, H″), (B), and (D, F, H) respectively

**FIGURE 5:**
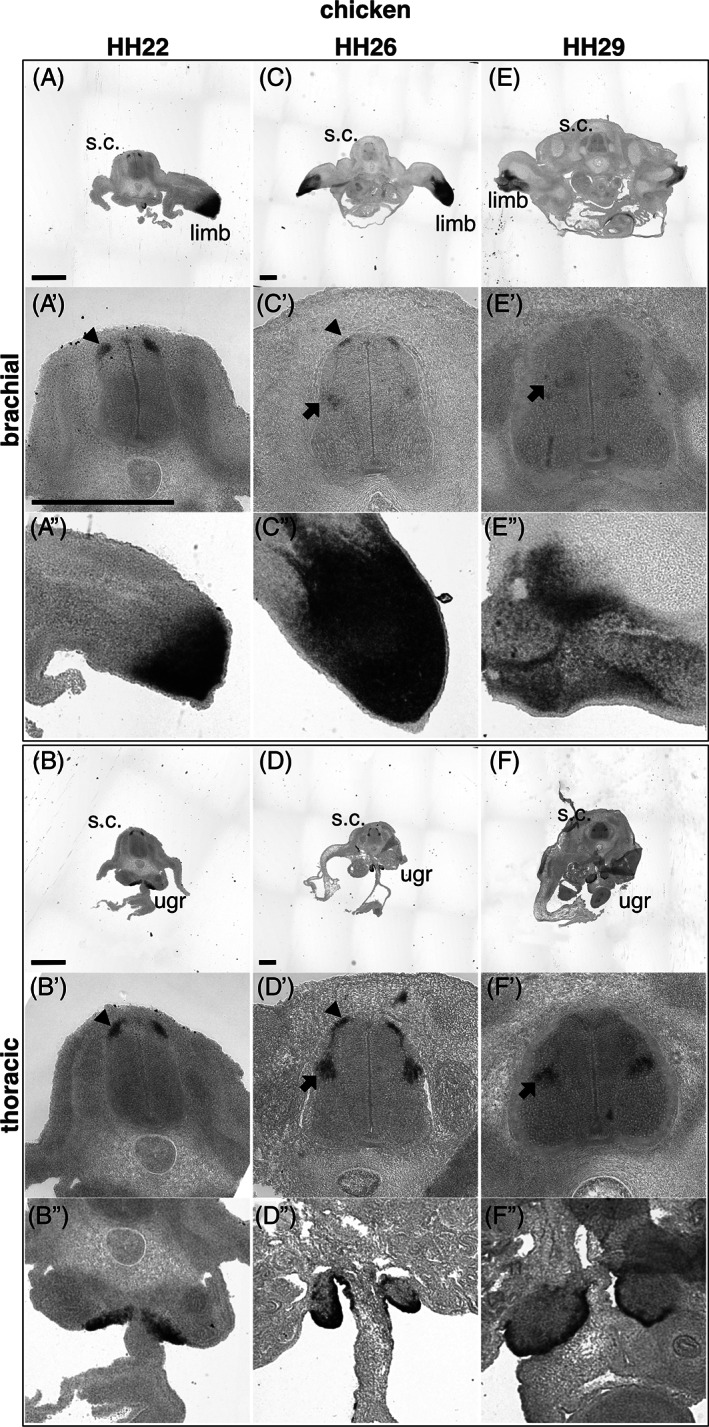
*Lhx9* is expressed in mid‐gestation chicken embryos. Brightfield images of brachial and thoracic transverse sections of chicken embryo at HH22 (A, A′, A″, B, B′, B″), HH26 (C, C′, C″, D, D′, D″) and HH29 (E, E′, E″, F, F′, F″) labeled by *in situ* hybridization with a probe against chicken *pan‐Lhx9*. The limb, spinal cord (s.c.), spinal cord dorsal mantel zone (black arrowhead), deep dorsal horn (black arrow) and urogenital ridge (ugr) are indicated. Higher magnification images are shown for the spinal cord (A′‐F′), the forelimb (A″, C″, E″) and urogenital ridge (B″, D″, F″). Representative images are shown. Three individual embryos were analyzed for each developmental age. Scale bars are 500 μm. The scale bars in A, A′, B, C, and D represent images in (A), (A′, A″, B′, B″, C′, C″, D′, D″, E′, E″, F′, and F″), (B), (C, E), and (D, F), respectively

First, *in situ* hybridization was used to determine the sum expression of *Lhx9* transcripts in mouse and chicken embryos using probes, which have that potential to recognize all known *Lhx9* splice variants, referred to as pan‐*Lhx9* (Figures 4 and 5). Since Lhx9 is known to influence embryo spinal neuron development during mid‐gestational time points, we focused on *Lhx9* expression during this period.^4^ We found that consistent with previous reports, *Lhx9* was expressed robustly in the spinal cord, limbs, and urogenital ridge at mid‐gestation ages in mouse and chicken embryos (Figures 4A‐H″ and 5A‐F″).^9^, ^13^, ^17^ Within the spinal cord, consistent with previous reports, *Lhx9* was expressed in the dorsal part of the gray matter in recently born dI1 neurons at E10.5/HH22 (Figures 4A,B,A',B' and 5A,B,A',B'). By E11.5/HH26, *Lhx9* remained expressed in this region at all levels examined and expression was also observed in a migrating stream of dI1 neurons, which at this age begin to settle in the deep dorsal horn (Figures 4C,D,C',D' and 5C,D,C',D'). At E12.5/HH29, *Lhx9* was robustly expressed in the deep dorsal horn at thoracic but weakly expressed at brachial levels of both mouse and chicken embryos (Figures 4E,F,E',F' and 5E,F,E',F'). In mouse E13.5 spinal cord, *Lhx9* was expressed almost exclusively in the deep dorsal horn (Figure 4G,H,G',H'). Taken together, these data confirm and extend previous findings that *Lhx9* is expressed in two major phases in dI1 neuron development, recently born dI1 neurons and neurons that have settled in the deep dorsal horn in both mouse and chick embryos (Figures 4 and 5).^4^, ^20^

**FIGURE 6:**
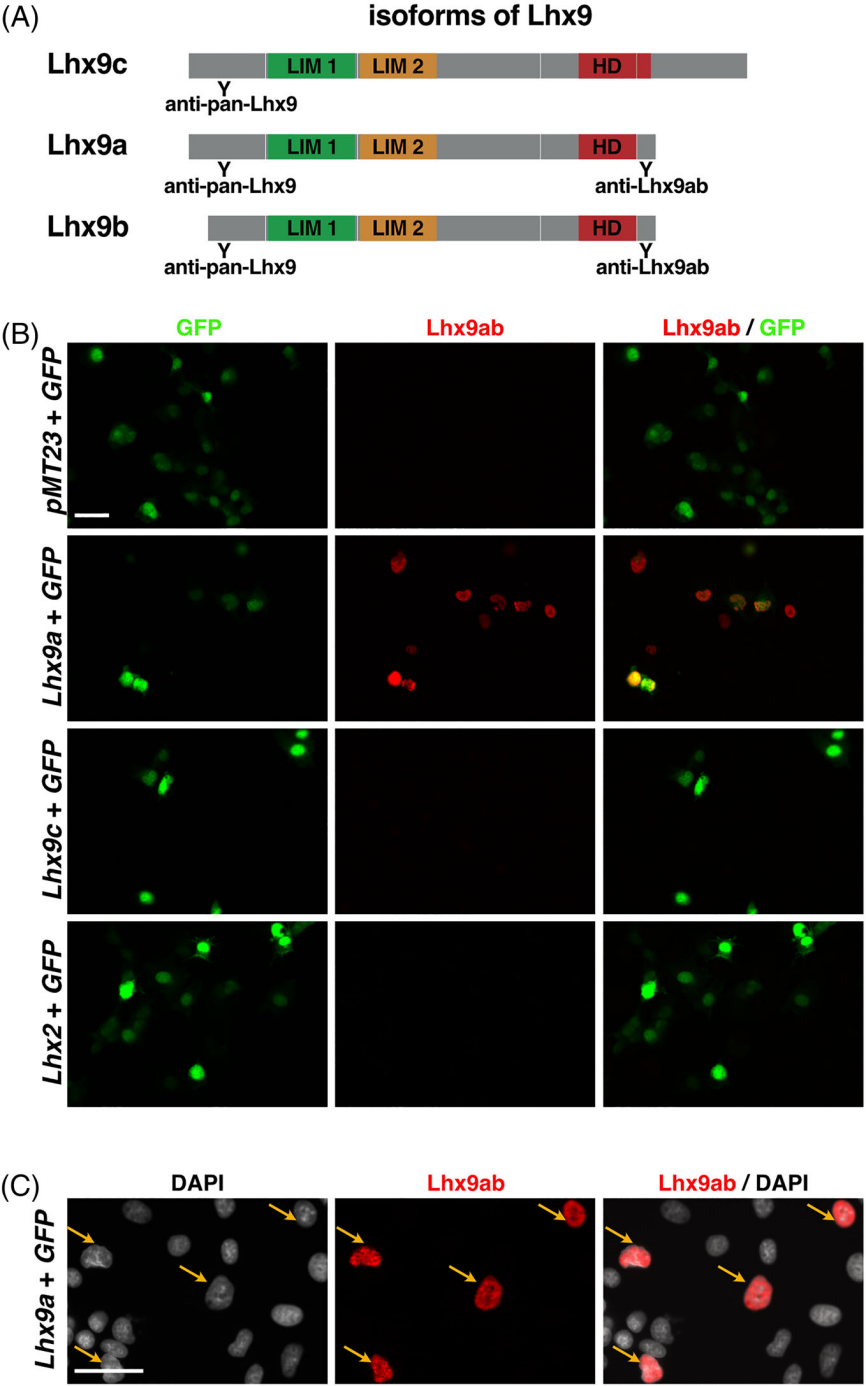
Validation of the specificity of the newly produced Lhx9ab antibody. (A) Schematics of the protein domain structures of Lhx9c (NP_001036042.1), Lhx9a (NP_001020736.1), and Lhx9b (NP_034844.1) are indicated. The LIM 1 (green) and LIM 2 (orange) domains and the homeodomain (HD red) are shown. The relative position targeted by the anti pan‐Lhx9 and anti Lhx9ab antibodies are indicated. (B) COS7 cells were transfected with either GFP‐expressing plasmid (*CMV‐GFP/pEGFP‐N2*) and control plasmid (*pMT23*) or GFP‐expressing plasmid (*CMV‐GFP/pEGFP‐N2*) and Lhx9a expressing or Lhx9c and GFP expressing plasmid or GFP‐expressing plasmid (*CMV‐GFP/pEGFP‐N2*) and Lhx2 expressing plasmid. Three technical repeats were performed; representative images are shown. (C) Higher magnification image of Lhx9ab labeling of overexpressed Lhx9a in COS‐7 cells shown in B counter stained with DAPI showing nuclear localization of the Lhx9a protein. Representative images are shown. Scale bars in B and C are 50 μm

**FIGURE 7:**
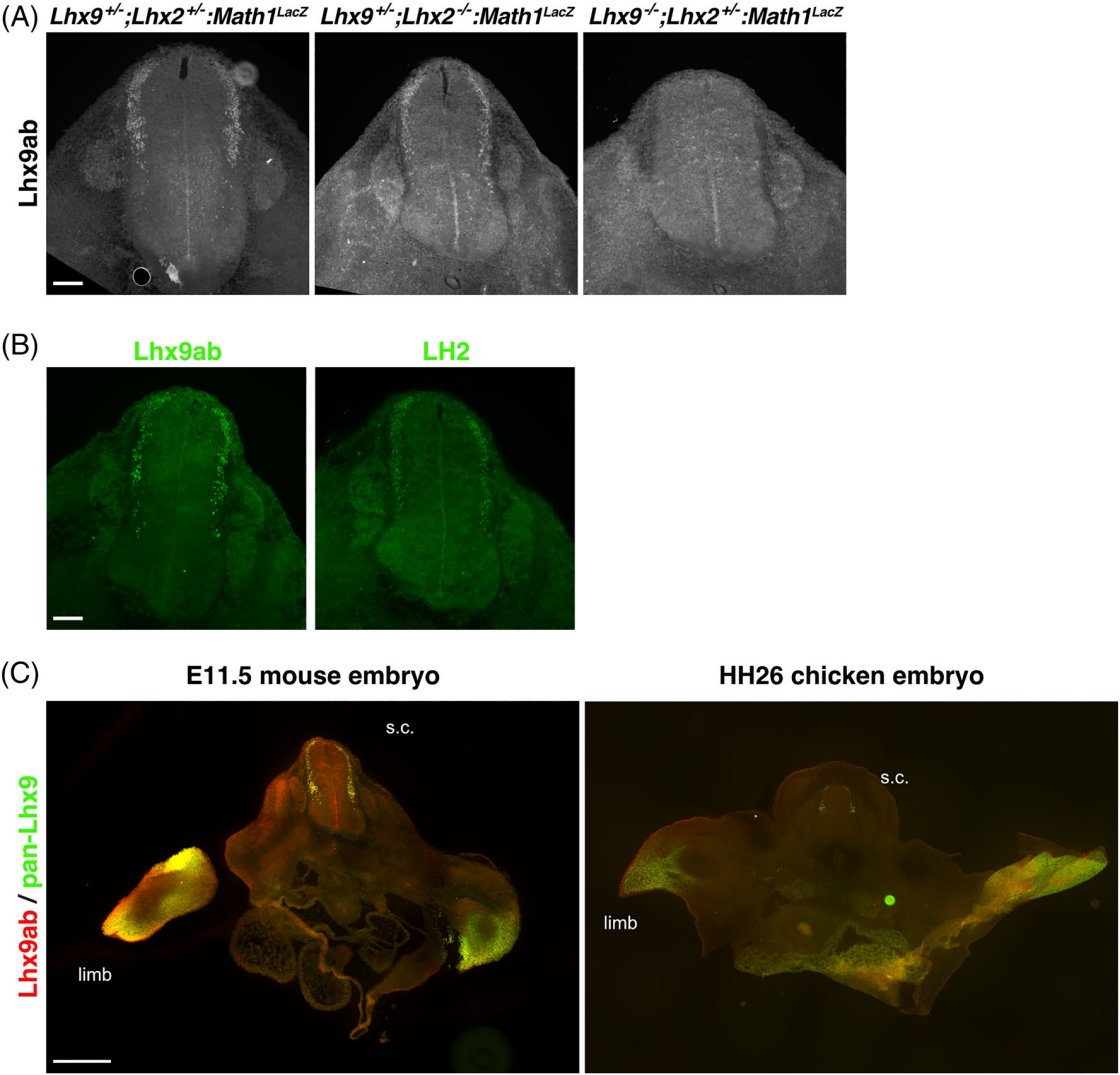
Lhx9ab protein expression in mid‐gestation embryo tissues. (A) Photomicrographs of transverse sections of E11.5 mouse embryonic spinal cord immunohistochemically labeled with the Lhx9ab antibody (white) from this study from control litermates (*Math1* ^*LacZ*^ or *Lhx9* ^*+/−*^ *;Lhx2* ^*+/−*^ *;Math1* ^*LacZ*^ heterozygote, n = 2), *Lhx2* mutant (*Lhx9* ^*+/−*^ *;Lhx2* ^*−/−*^ *;Math1* ^*LacZ*^, n = 3), *Lhx9* mutant (*Lhx9* ^*−/−*^ *;Lhx2* ^*+/−*^ *;Math1* ^*LacZ*^; n = 2) and *Lhx2:Lhx9* double mutants (*Lhx9* ^*−/−*^ *;Lhx2* ^*−/−*^ *;Math1* ^*LacZ*^; n = 2) mouse embryos. (B) Serial transverse sections of E11.5 brachial spinal cord labeled with antibodies against Lhx9ab and LH2 (which labels both Lhx9 and Lhx2^6^) showing total distribution of the dI1 neuron population at this age (n = 3). (C) Transverse sections of brachial level embryonic mouse and chicken embryos immunofluorescently labeled with antibodies against pan‐Lhx9 (green) and Lhx9ab (red). Representative images are shown. Scale bars are 100 μm in A and B and 500 μm in C

**FIGURE 8:**
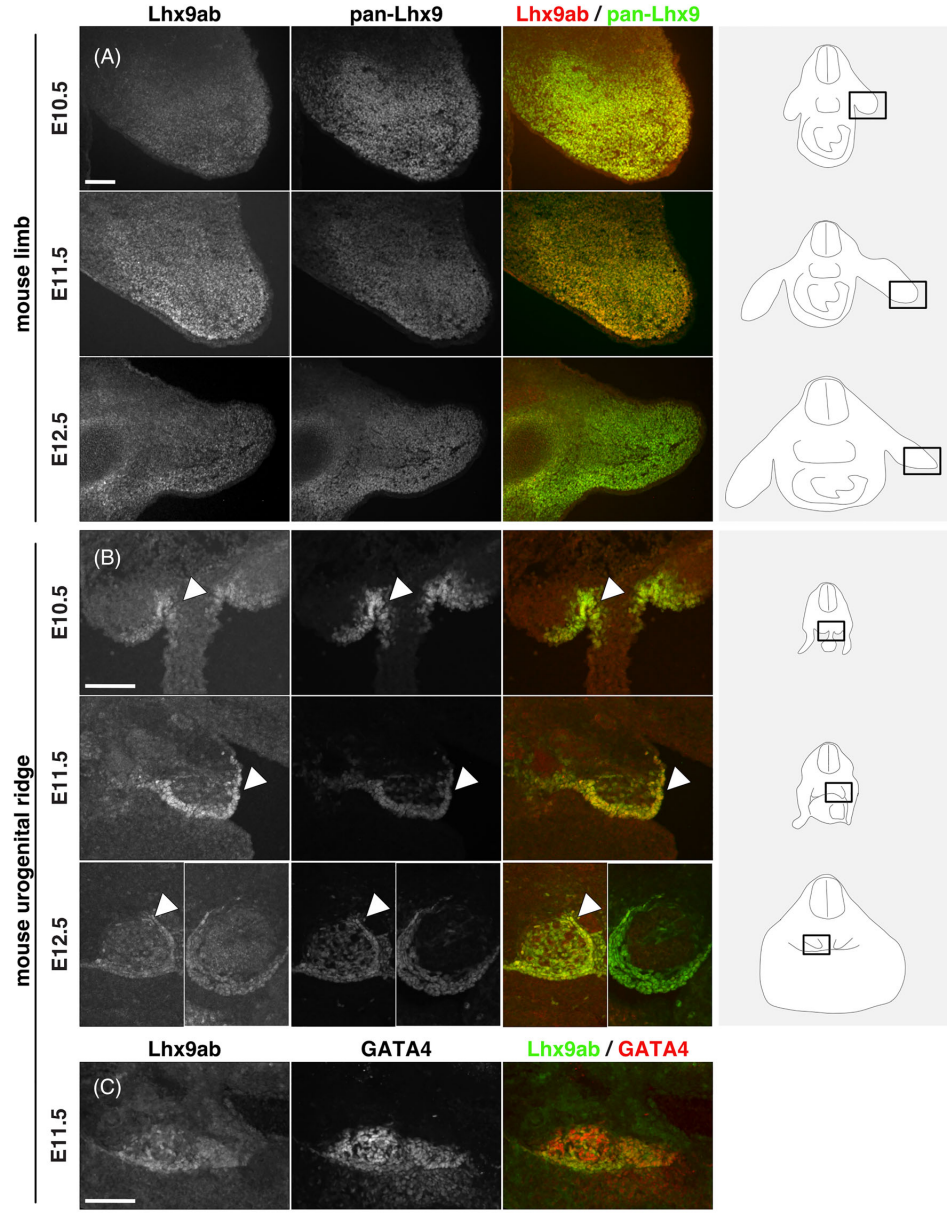
Lhx9 and Lhx9ab expression in limbs and urogenital ridge of mid‐gestation mouse embryos. Transverse sections of mouse embryos labeled with antibodies against pan‐Lhx9 (white or green) and Lhx9ab (white or red) at E10.5, E11.5, and E12.5 of developing forelimb in brachial sections (A) and urogenital ridge and developing gonad in thoracic sections (B). The arrowhead in B points to the urogenital ridge/developing gonad. By E12.5, variation in the expression of Lhx9ab was observed consistent with gonadal differentiation therefore two contrasting mouse E12.5s sample are shown separated by a white line. (C) Thoracic sections were also co‐labeled with antibodies against Lhx9ab and GATA4, another known marker of the urogenital ridge. Three to four embryos were analyzed for each developmental age. Representative images are shown. Scale bar in A, B, and C are 100 μm and applies to all images of same magnification

**FIGURE 9:**
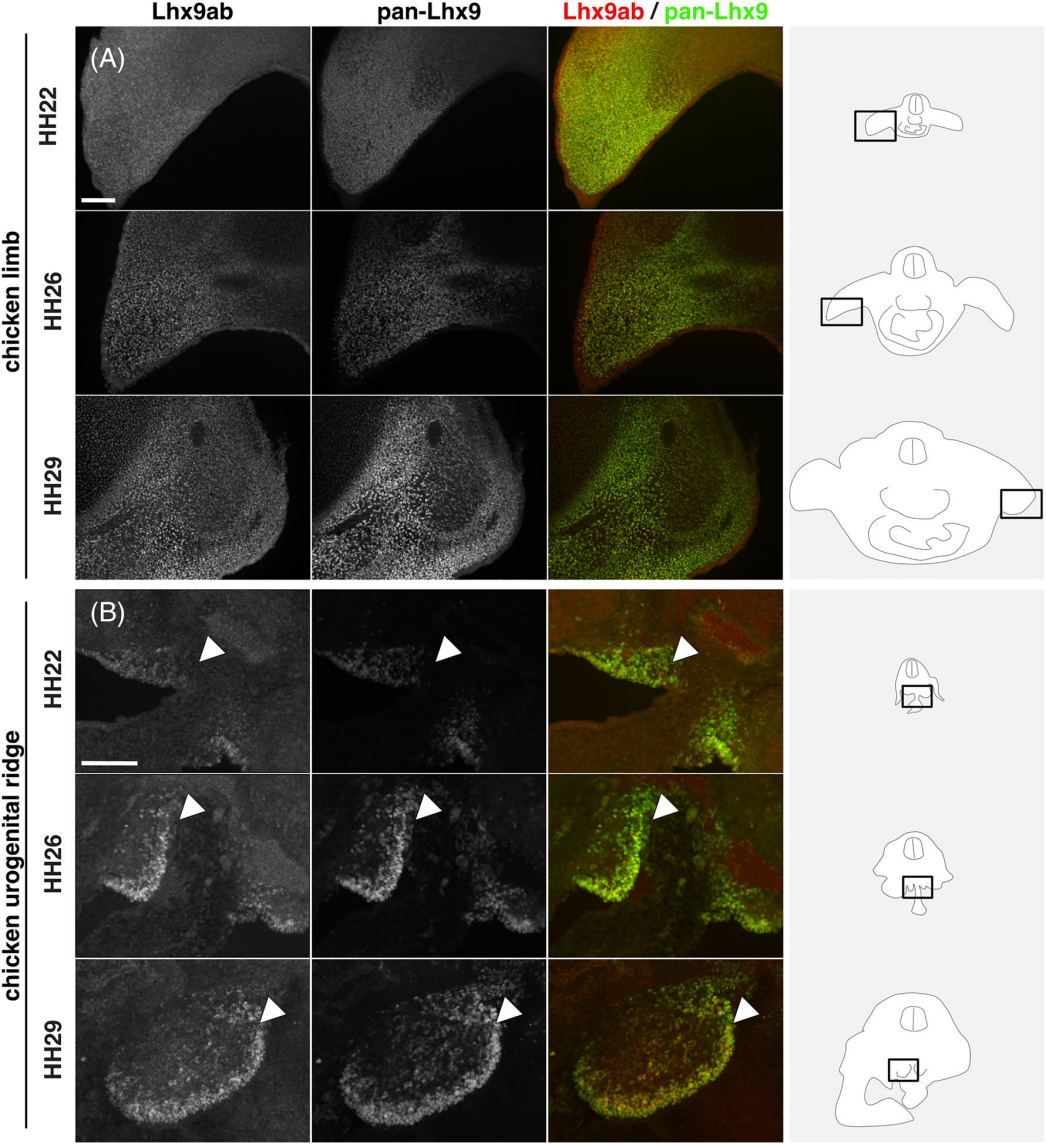
Lhx9 and Lhx9ab expression in limbs and urogenital ridge of mid‐gestation chicken embryo tissues. Transverse sections of chick embryo labeled with antibodies against pan‐Lhx9 (white or green) and Lhx9ab (white or red) at HH22, HH26, and HH29 of developing forelimb in brachial sections (A) and urogenital ridge and developing gonad in thoracic sections (B). The arrowhead in B points to the urogenital ridge/developing gonad. Three to four embryos were analyzed for each developmental age. Representative images are shown. Scale bar in A and B is 100 μm and applies to all images of same magnification

**FIGURE 10:**
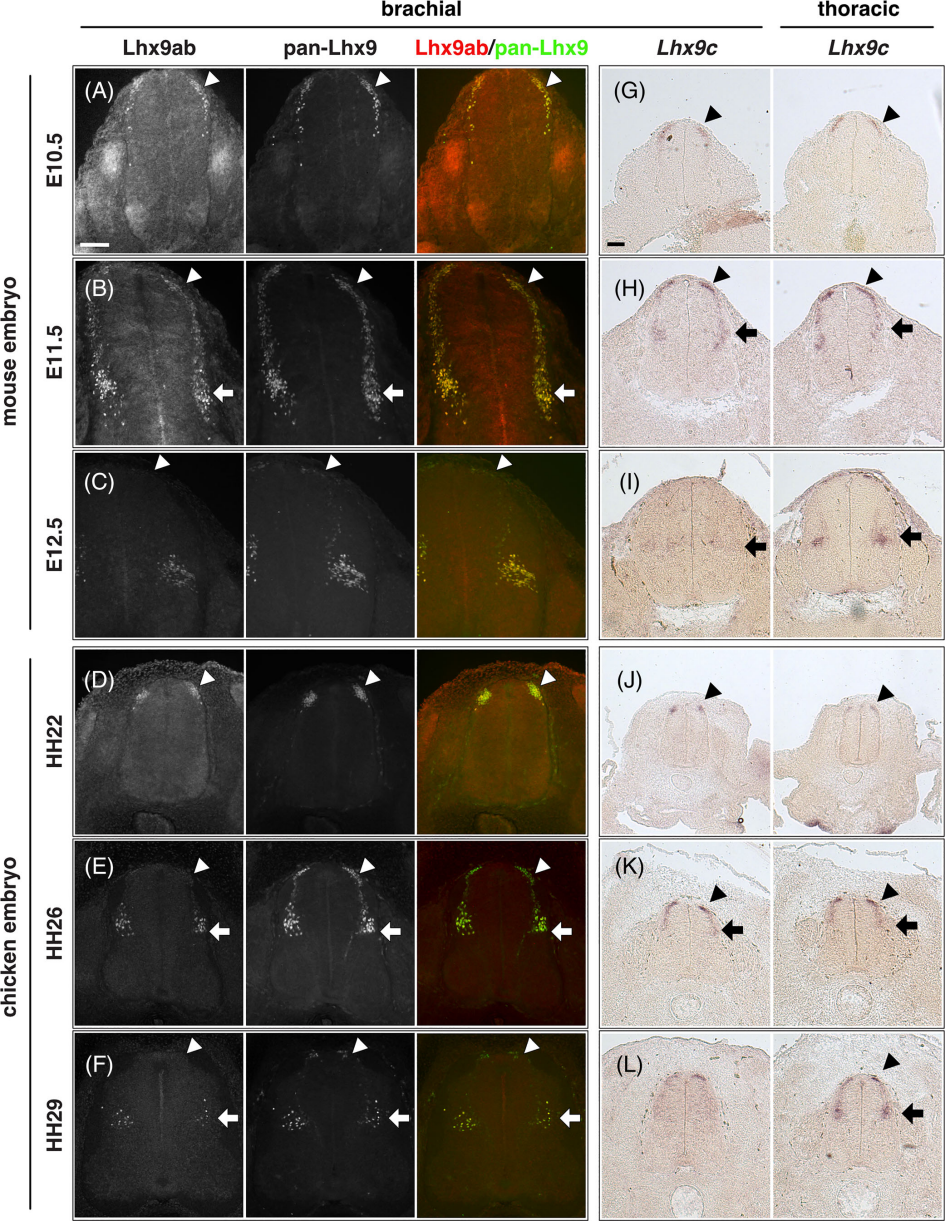
*Lhx9c* and Lhx9ab are differentially expressed during development of the spinal cord. Transverse sections of embryonic mouse and chicken embryos immunofluorescently labeled with pan‐Lhx9 (green or white) and Lhx9ab (red or white) antibodies (A‐F) or labeled by *in situ* hybridization with *Lhx9c* mRNA probe (G‐L) are shown. (A ‐ F) Brachial level spinal cord sections for mouse embryonic tissue at E10.5 (A), E11.5 (B), E12.5 (C) and chicken embryos at HH22 (D), HH26 (E), and HH29 (F) are shown. (G‐L) *In situ* hybridization specifically targeting the canonical variant of the *Lhx9* gene (*Lhx9c*) is shown at brachial and thoracic levels of the spinal cord of mouse embryonic tissue at E10.5 (G), E11.5 (H), E12.5 (I), and chicken embryos at HH22 (J), HH26 (K), and HH29 (L). Arrowheads indicate position of dorsal mantle zone and horizontal arrows indicate deep dorsal horn, respectively. Three to five embryos were analyzed for each developmental age; representative images are shown. Scale bar in A and G are 100 μm and apply to all images of the same magnification

While this analysis determined the overall comparative spatiotemporal distribution of *Lhx9*, it did not determine whether canonical or noncanonical *Lhx9* variants were expressed (Figures 4 and 5). To this end we generated an antibody, which exclusively recognized the alternative C‐terminal sequence shared by Lhx9a and Lhx9b (referred to here as Lhx9ab; Figure 6A). The specificity of this Lhx9ab antibody was carefully verified. First Lhx9ab antibody recognized Lhx9a overexpressed in cell culture whereas it did not recognize overexpressed Lhx9c, Lhx2, or GFP proteins (Figure 6B). We noted that in this context, the labeling of the overexpressed Lhx9a protein was nuclear (Figure 6C). The specificity of the antibody was further tested in *Lhx9* knockout and control mouse embryos. Lhx9ab antibody labeling was detected in the spinal cord of mouse embryos null for the either the *Lhx9* paralogue *Lhx2*, embryos heterozygote for *Lhx2* or *Lhx9* or wild‐type but was not detected in embryos, which were null for *Lhx9* (Figure 7A). Finally, labeling of the spinal cord with the newly created Lhx9ab antibody was detected in an overlapping region to the distribution of a previously characterized dI1 antibody marker LH2 which recognizes both Lhx2 and Lhx9 (Figure 7B).^6^ Together, this demonstrated that the newly generated Lhx9ab antibody was specific for the intended target. The expression of this Lhx9ab antibody was first compared with a previously generated Lhx9 antibody that recognized all known splice variants (referred to as pan‐Lhx9; Figure 6A).^21^ At the ages and species examined, we observed labeling of both pan‐Lhx9 and Lhx9ab antibodies in a number of regions within the body, notably the spinal cord, urogenital ridge and developing limbs (Figure 7C). Outside the nervous system, in mouse/chicken E10.5/HH22 embryos, pan‐Lhx9 and Lhx9ab antibodies were clearly detected in the developing limbs and in the urogenital ridge (Figure 8A‐C and 9A,B). At E11.5/HH26, although pan‐Lhx9 and Lhx9ab antibodies were both present in the developing limbs it appeared that Lhx9ab was more intensely labeled in the distal limb compared with the pan‐Lhx9 labeling suggesting that Lhx9 and Lhx9ab protein were differentially distributed (Figures 8A and 9A). At E10.5/HH22 and E11.5/HH26 expression of both panLhx9 and Lhx9ab were detected in the urogenital ridge, which was confirmed by expression in the same region with GATA4, a known marker of the urogenital ridge (Figures 8B,C and 9B). By E12.5 differences in expression of Lhx9ab between samples was observed within the developing gonad (Figure 8B). Within the spinal cord at E10.5/HH22 we observed expression of both pan‐Lhx9 and Lhx9ab in newly born dI1 neurons (Figure 10A,D, Table 1). Strikingly, within the spinal cord we observed a clear distinction of labeling between Lhx9ab and pan‐Lhx9 in chicken HH26/HH29 and mouse E11.5/E12.5 where pan‐Lhx9 was expressed more broadly than Lhx9ab (compare regions indicated by arrows and arrowheads in Figure 10B,C,E,F and summarized in Table 1). At chicken HH26, pan‐Lhx9 was expressed in newly generated dI1 neurons at the dorsal spinal cord and in dI1 neurons in a migrating stream as they migrated to the deep dorsal horn (Figure 10E). In contrast, Lhx9ab was expressed predominantly in the deep dorsal horn (Figure 10E). This was also observed for mouse E11.5 embryos, however in that case a dorsoventral difference in the distribution between pan‐Lhx9 and Lhx9ab was observed in about half the embryos examined suggesting that this could be a time dependent feature (Figure 10B). By HH29/E12.5 in the spinal cord of chicken/mouse embryos Lhx9ab expression was exclusively found in the deep dorsal horn whereas pan‐Lhx9 was also detected in the dorsal spinal cord in newly generated dI1 neurons (see arrowhead in Figure 10C,F).

**TABLE 1:** Summary of panLhx9 and Lhx9ab antibody labeling and *Lhx9c* *in situ* mRNA expression shown in this study

	Marker	Mouse			Chicken		
		E10.5	E11.5	E12.5	HH22	HH26	HH29
Recently born dI1 neurons in the dorsal spinal cord	panLhx9	++	++	+	++	++	+
	Lhx9ab	++	+	−	++	+	−
	*Lhx9c* mRNA	++	++	−	++	++	+
Deep dorsal horn	panLhx9	−	++	+++	−	++	+++
	Lhx9ab	−	++	+++	−	++	+++
	*Lhx9c* mRNA	−	+	+	−	+	+

*Note*: A summary of panLhx9 and Lhx9ab immuno labeling (Figures 8, 9, 10, 11, 12, 13) and *Lhx9c in situ* hybridization labeling (Figure 10) is shown. The relative expression level in the brachial spinal cord in either the dorsal mantel zone or deep dorsal horn is indicated: − (blue, no expression detected), +, ++, or +++ to indicate increasing levels of expression (increasing intensity in red represents the reletive expression from light red (low expression) to dark red (strongest expression)).

Overall, pan‐Lhx9, which labels all Lhx9 variants, was expressed at all stages of dI1 neuron development analyzed: in neurons as they delaminate from the ventricular zone, in migrating neurons and in maturing neurons as they settle in the deep dorsal horn. In contrast, Lhx9ab appeared to be strongly expressed only in first wave of dI1 neurons generated at E10.5/HH22 and those settling in the deep dorsal horn (Figure 10 and Table 1). Taken together, this supported the notion that Lhx9c (delimited by pan‐Lhx9^+^/Lhx9ab^−^ expression) was expressed in newly generated dI1 neurons whereas noncanonical Lhx9ab was expressed in the first wave of newly generated dI1 neurons and subsequently became expressed in later development as the neurons started to mature and settle in the deep dorsal horn.

In the above investigation, the analysis of Lhx9c expression was based on defining regions that expressed panLhx9 where Lhx9ab labeling was absent. Consequently, this analysis did not provide information whether Lhx9c was co‐expressed in the deep dorsal horn together with Lhx9ab or not. Therefore, we next generated a riboprobe that specifically recognized *Lhx9c* but not *Lhx9ab* transcripts and examined its expression in mouse and chicken embryonic spinal cord (Figure 10G‐L). We found that at HH22/E10.5 and HH26/E11.5 similar to the pan‐Lhx9 antibody, *Lhx9c* probe was observed most strongly in the recently generated dI1 neurons in the dorsal spinal cord (Figure 10G,H,J,K, Table 1). At mouse HH29/E12.5 *Lhx9c* was detected in the deep dorsal horn, most strongly in the thoracic region (Figure 10I,L). These data were consistent with the immunohistochemical data, supporting the notion that the canonical *Lhx9* transcripts were expressed in the first wave of dI1 neurons generated. Taken together, this implied that both canonical and noncanonical Lhx9 splice variant proteins had a dynamic temporally precise and partly exclusive and partly overlapping expression pattern.

### 2.3 Noncanonical Lhx9ab is expressed at key developmental choice points in developing dI1 neurons

**FIGURE 11:**
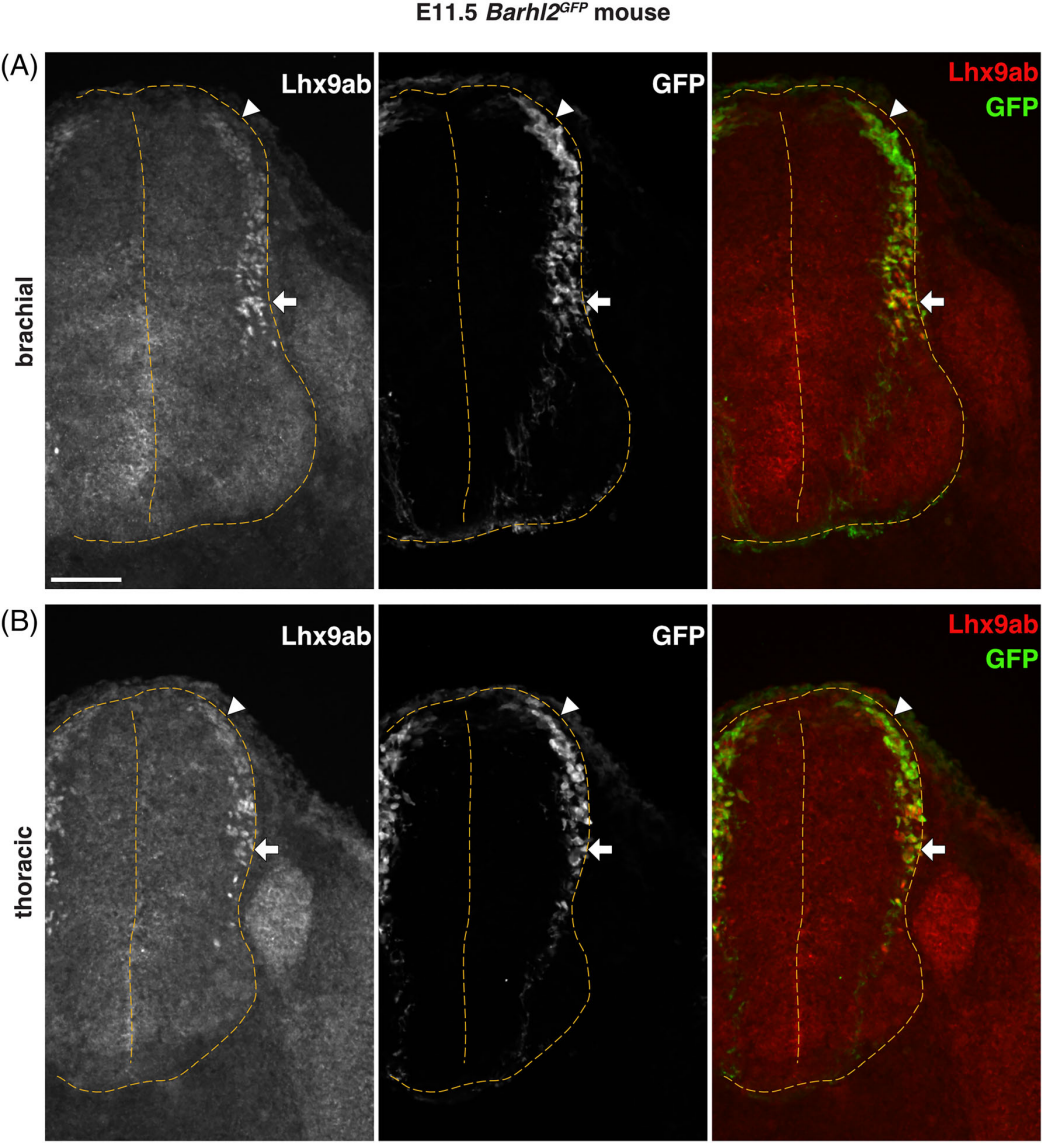
Lhx9ab is dynamically expressed in maturing dI1 neurons in the E11.5 mouse spinal cord. Photomicrographs of transverse spinal cord sections of E11.5 *Barhl2* ^*GFP*^ embryos (n = 4 embryos) immunofluorescently labeled with GFP (green or white) and Lhx9ab (red or white) shown as single channel images and merged images. Brachial (A) and thoracic (B) levels are shown. Arrowheads indicate the dorsal mantle zone and horizontal arrows indicate neurons settling in the deep dorsal horn. The outline and midline of the spinal cord are delineated with yellow dashed lines. Representative images are shown. The scale bar is 100 μm and represents all images

**FIGURE 12:**
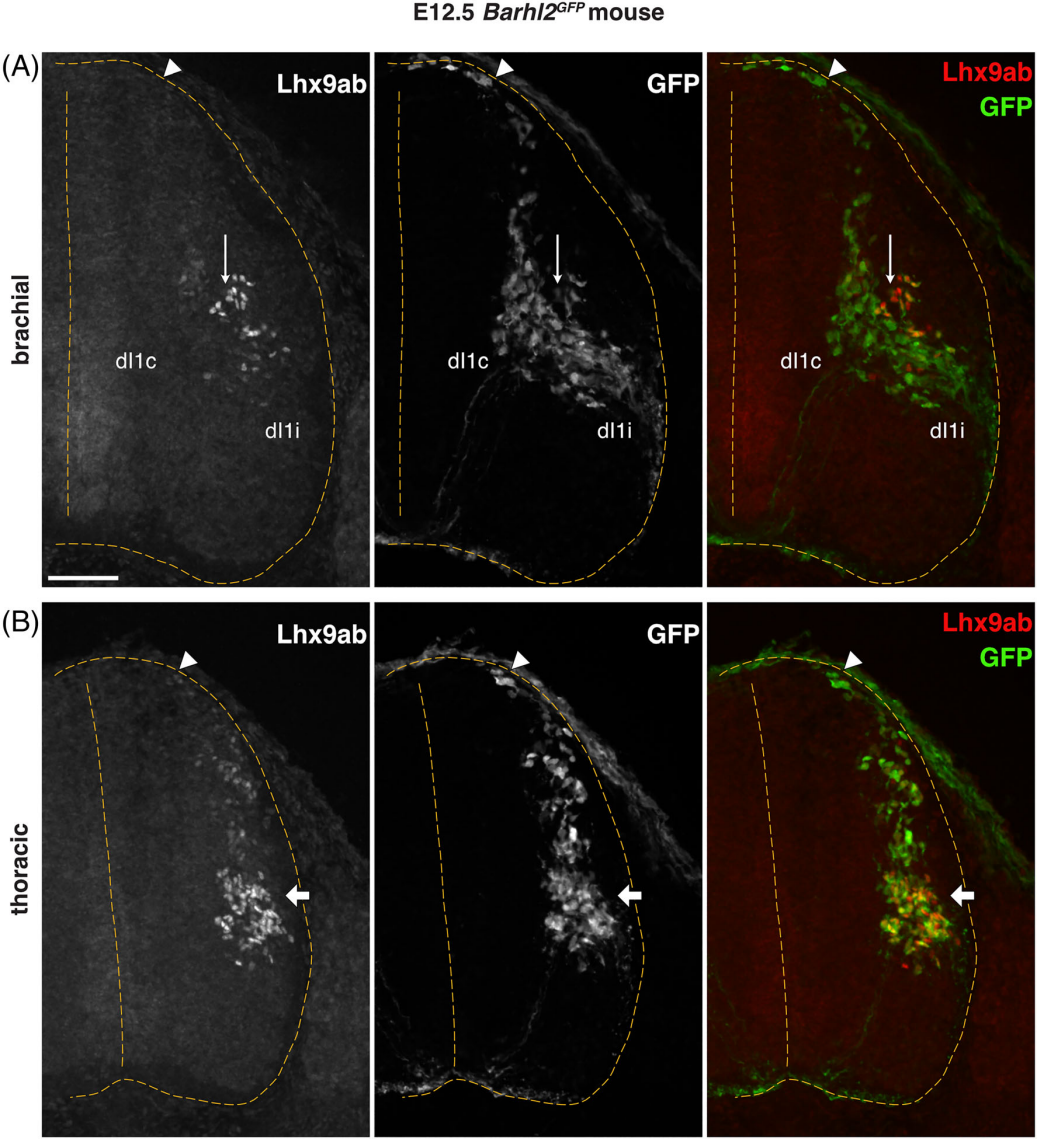
Lhx9ab is dynamically expressed in maturing dI1 neurons in the E12.5 mouse spinal cord. Photomicrographs of transverse spinal cord sections of E12.5 *Barhl2* ^*GFP*^ embryos (n = 7 embryos) immunofluorescently labeled with GFP (green or white) and Lhx9ab (red or white) shown as single channel images and merged images. Brachial (A) and thoracic (B) levels are shown. The position of the arrowhead indicates dorsal mantle zone, the horizontal arrow indicates neurons settling in the deep dorsal horn and the vertical arrow indicates neurons dorsal to the ipsilateral (dI1i) and contralateral (dI1c) dorsal spinal interneuron populations. The outline and midline of the spinal cord are delineated with yellow dashed lines. Representative images are shown. The scale bar is 100 μm and represents all images

**FIGURE 13:**
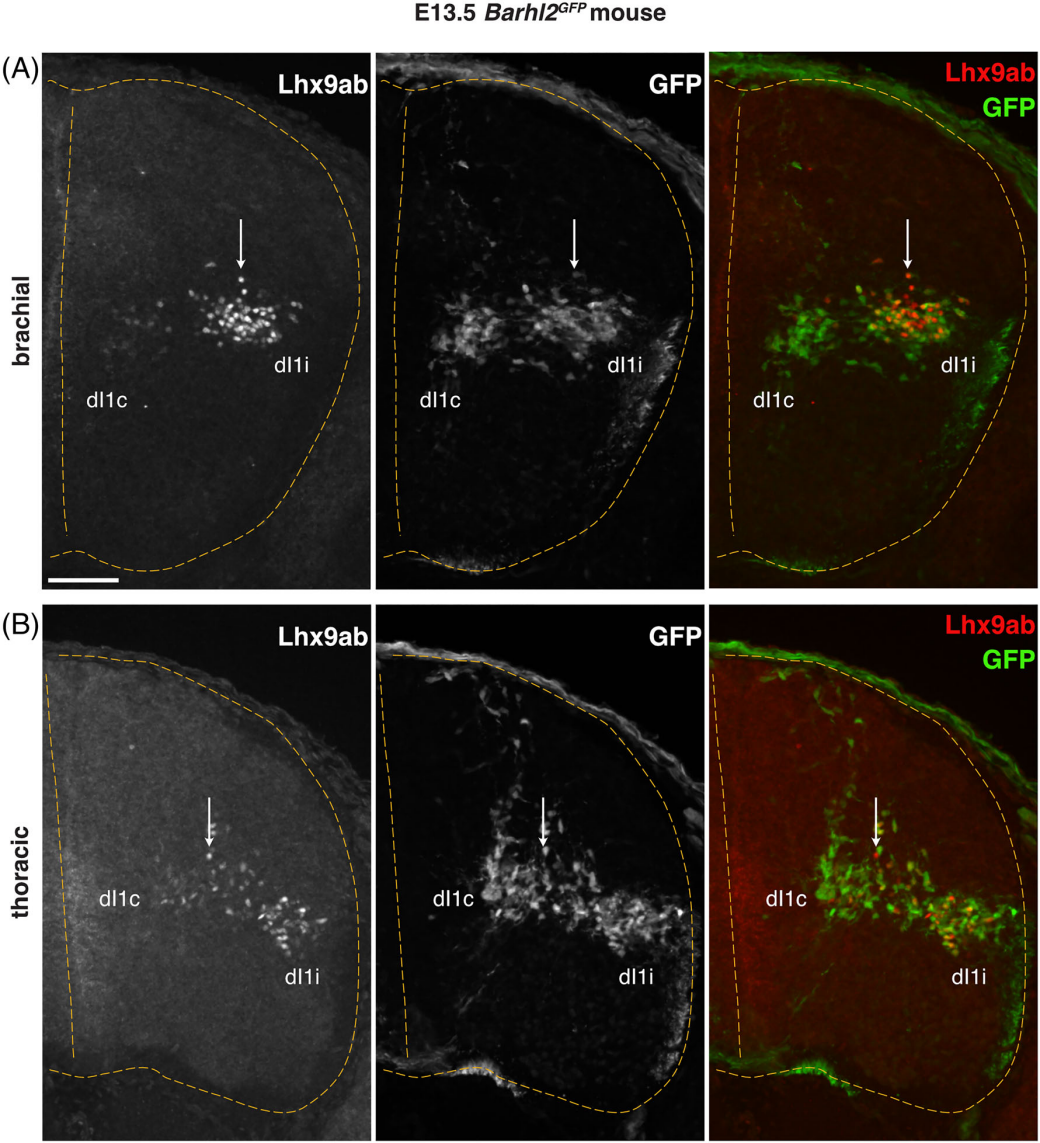
Lhx9ab is dynamically expressed in maturing dI1 neurons in the E13.5 mouse spinal cord. Photomicrographs of transverse spinal cord sections of E13.5 *Barhl2* ^*GFP*^ embryos (n = 4 embryos) immunofluorescently labeled with GFP (green or white) and Lhx9ab (red or white) both as single channel images and merged images. Brachial (A) and thoracic (B) levels are shown. The vertical arrow indicates neurons dorsal to the ipsilateral (dI1i) and contralateral (dI1c) projecting dorsal spinal interneuron populations. The outline and midline of the spinal cord are delineated with yellow dashed lines. Representative images are shown. The scale bar is 100 μm and represents all images

The dynamic spatiotemporal expression pattern of Lhx9ab protein suggested that it was expressed at key developmental choice points in the development of dI1 neurons. In order to examine this further we took advantage of a mouse transgenic model, *Barhl2* ^*GFP*^, which genetically labels spinal cord dI1 neurons with GFP permitting the anatomical tracing of dI1 neurons.^4^ dI1 neurons are derived from a common precursor domain and give rise to anatomically distinct populations including dI1 commissural (dI1c) and dI1 ipsilateral (dI1i) projecting neurons.^4^ Using *Barhl2* ^*GFP*^ transgenic mouse embryos, we examined embryonic stages before, during and after the anatomical divergence of dI1c and dI1i neurons (E11.5‐E13.5) to determine whether Lhx9ab expression was correlated with these major developmental choice points. In E11.5 *Barhl2* ^*GFP*^ embryos, we observed weak expression of Lhx9ab in ventrally migrating dI1 neurons (Figure 11). At brachial levels of the spinal cord, which is more developmentally advanced than thoracic levels, in some embryos we observed a gradual downregulation of Lhx9ab protein in dorsally located dI1 neurons and an increase in Lhx9ab protein in GFP^+^ dI1 neurons that had reached the deep dorsal horn. This was consistent with expression of Lhx9ab in dI1c neurons (Figure 11). By E12.5 a striking and dynamic shift of Lhx9ab labeling in maturing dI1 neurons was observed (Figure 12). In thoracic levels of E12.5 *Barhl2* ^*GFP*^ embryos, similar to brachial levels at E11.5, strong Lhx9ab expression was observed in GFP^+^ dI1 neurons that had migrated to the deep dorsal horn whereas the expression was relatively weak in more dorsally located GFP^+^ neurons (Figure 12B). In contrast, in the more developmentally advanced brachial levels of E12.5 *Barhl2* ^*GFP*^ embryos, Lhx9ab was strongly expressed in a subset of neurons in a dorsal band of GFP^+^ neurons in the lateral deep dorsal horn, consistent with expression in dI1i neurons with very low levels detected in in medially located GFP^+^ dI1c neurons (Figure 12A vertical arrow). Very low levels were detected in medially located GFP^+^ dI1c neurons at brachial levels (Figure 12A). Similarly, in E13.5 *Barhl2* ^*GFP*^ embryos, Lhx9ab protein was expressed in laterally positioned dI1i neurons whereas it was barely detectable in the medially positioned dI1c neurons (Figure 13 vertical arrow). Overall, these data revealed that within the developing spinal cord, Lhx9ab protein was expressed in a temporally dynamic manner consistent with developmental choice points in dI1 neuron anatomical divergence.

## 3 DISCUSSION

The aim of this study was to examine the protein distribution of the noncanonical Lhx9 variants using an antibody tool designed to selectively detect them. The most important finding of this study was the demonstration that noncanonical Lhx9ab variants were translated and dynamically expressed in developing mouse and chick embryos at key developmental choice points. These observations, taken together with the high evolutionary conservation and the important structural differences between variants, supports the notion that the expression of canonical vs noncanonical Lhx9 variants plays a fundamental role in development.

### 3.1 Lhx9ab in the developing spinal cord

We have previously shown in the developing mouse spinal cord that Lhx9 and its paralogue Lhx2 act redundantly to regulate dI1 commissural (dI1c) neuron trajectory. This is achieved by Lhx2 and Lhx9 binding to palindromic DNA sequences to elicit expression of the commissural neuron guidance receptor *Robo3* (Figure 1A). The results from that study pointed to a canonical LIM‐HD transcriptional function of Lhx9 and Lhx2 in the regulation of *Robo3*.^4^ In the developing spinal cord, the commissural axon phenotype in *Lhx2:Lhx9* mutant embryos was predominantly observed at E10.5 and E11.5 suggesting it involved the first‐born dI1 neurons.^4^ Consistent with this we showed here that *Lhx9c* mRNA is expressed in the first wave of dI1 neurons born at E10.5/HH26 and E11.5/HH26 mouse/chicken embryos. Interestingly of the recently born dI1 neurons, in addition to *Lhx9c* we also noted clear expression of noncanonical Lhx9ab protein at E10.5/HH22 mouse/chicken embryos, which suggests a role for Lhx9ab variants in the first‐born dI1 neurons. This expression in newly generated dI1 neurons was not apparent in later born neurons. Rather, at later developmental time points, we noted that as dI1 neurons migrated ventrally they expressed higher levels of Lhx9ab, which then became enriched in neurons located laterally in the deep dorsal horn. As dI1 neurons mature and migrate ventrally they form two distinct anatomical populations dI1 commissural neurons (dI1c), which project commissurally and dI1 ipsilateral (dI1i) neurons, which project axon ipsilaterally.^4^ The expression of Lhx9ab in this lateral position indicates that Lhx9ab variants could play a role in dI1i development. However, the role of Lhx9ab variants in the spinal cord or elsewhere remains unknown. In addition to the spinal cord study described above, defects in gonad, limb, and proepicardial organ development have also been observed in *Lhx9* and *Lhx2:Lhx9* mutant or *Lhx9* knockdown embryos, respectively.^4^, ^13^, ^16^, ^22^ In these studies, all *Lhx9* transcript variants are affected and therefore the function of Lhx9ab variants was not examined.

While not much work has been done specifically on the mechanism of action of Lhx9c, its classical canonical secondary structure taken together with the fact that in a biochemical assay Lhx9c can bind DNA containing a well‐established LIM‐HD binding sequence and linker protein suggest that Lhx9c acts as a classical canonical LIM‐HD transcription factor (Figure 1A).^4^, ^8^ Compared with its canonical Lhx9 counterpart, the noncanonical Lhx9ab variants have three notable features that could affect their function. The third helix of the homeodomain and the canonical sequence C‐terminal to the homeodomain are both missing and there is an addition of a C‐terminal sequence that is unique to the Lhx9ab variants. One hypothesis has suggested that the lack of the third helix of the Lhx9 homeodomain in the noncanonical Lhx9ab variants may render them as dominant negative molecules that suppress LIM‐HD transcription factor signalling.^8^, ^10^, ^11^ This idea stems from the knowledge that Lim Domain Only (LMO) family members, which have a LIM‐domain but entirely lack the homeodomain serve a dominant negative function to LIM‐HD transcription factors.^23^ Supporting this, Lhx9a was unable to bind to a known LIM‐HD transcription factor DNA sequence in biochemical experiments in vitro.^8^ Another study showed that Lhx9a could bind to the LIM‐HD transcription factor Isl1 when both were over expressed in cultured cells.^12^ While these lines of reasoning support a dominant negative function of Lhx9ab, explicit experimental evidence for this is lacking and indeed several lines of evidence suggest otherwise. In a cell culture assay, neural differentiation was increased when transfected with a canonical *Lhx9* expression construct.^8^ Expression of Lhx9a together with Lhx9c in this context did not result in decreased neural differentiation, but indeed instead Lhx9a elicited neural differentiation at about the same rate as canonical Lhx9.^8^ This suggested that Lhx9a did not have a dominant negative function. It is interesting to note that unlike LMO proteins Lhx9ab still retains a significant proportion of the homeodomain, albeit truncated. Further, the noncanonical Lhx9 variants also gain a different C‐terminal sequence which may serve a yet to be discovered function. For example, it has been shown for another LIM‐HD transcription factor, Isl1, the region C‐terminal to the homeodomain has an important function in binding other LIM‐domains, in essence acting as a self‐contained linker protein to interact with other LIM‐HD transcription factors.^24^ Thus, the possibility remains open that the Lhx9ab C‐terminal domain has an important function that may serve a Lhx9ab specific function which therefore remains yet‐to‐be‐determined.

Overall, while the evidence supports the notion that canonical and noncanonical Lhx9 transcription factors have differential DNA binding properties, no good evidence exists for its function and its role in dI1 neuron development therefore remains to be determined.

### 3.2 mRNA stability/availability and translation

Using riboprobes and antibodies that recognize all splice variants we observed differences in mRNA and protein expression where the protein expression perdures longer. This could be as a consequence of method sensitivity levels, endogenous transcript stability or translation regulation. A previous study has suggested, based on mRNA expression, that *Lhx9a* expression is expressed in later development but to a lesser extent in early development.^10^ On the contrary, our study of the protein distribution suggested that within the spinal cord, noncanonical Lhx9ab variant are expressed biphasically: First together with *Lhx9c* in the initial wave of dI1 neurons born at E10.5/HH22, E11.5/HH26, and subsequently as the neurons mature and settle in the deep dorsal horn. In other studies, splice variants of *Lhx9* have been shown to be expressed in various species at the transcript level.^12^, ^13^, ^14^, ^15^, ^16^ In attempting to discern the protein expression of the Lhx9a variant in mouse heart development, one study used an antibody which recognized all known splice variants of Lhx2 and Lhx9 together with the lack of detection of canonical *Lhx9c* mRNA at the stage examined.^12^ However, given the framework of the sensitivity levels for mRNA and protein expression our study suggests that variant‐selective antibodies should be used to make formal conclusions of variant expression. In this respect, a caveat of our study was that since an Lhx9 canonical specific antibody was not available, the determination of Lhx9c expression relied on analysis of either cells that were pan‐Lhx9^+^/Lhx9ab^−^ by immunohistochemistry or *Lhx9c* mRNA positive by *in situ* hybridization and thus, there may be other regions of Lhx9c protein expression that were not described here. Therefore, tools that selectively label transcript variants as done in this study are fundamental to understanding pathway molecular mechanisms and differential splice variant functions.

### 3.3 Overall conclusion

Different splice variants have been predicted widely among mammalian LIM‐HD transcription factors. Of the LIM‐HD transcription factors, Lhx9 and Lhx8 (previously called Lhx7) are currently reported to have splice variants that result in a truncated homeodomain.^25^ Of note, the *Lhx9ab* alternative spicing of the *Lhx9* gene is highly conserved among a wide range of vertebrates, indicating that it is an important feature in vertebrate development. Taken together, the wider implications are that noncanonical splice variants of Lhx9 are likely to have profound biological functions. More broadly, understanding how splice variation influences molecular mechanisms is likely to reveal powerful mechanisms for developmental regulation. Understanding the temporal expression dynamics of the variants of gene families such as the study presented here is a key entry point to understanding what influence respective splice variants have.

## 4 EXPERIMENTAL PROCEDURES

### 4.1 Bioinformatic analysis

Gene, transcript, and protein sequences were obtained from the National Center for Biotechnology Information (NCBI) database.^26^ Multiple sequence alignments were generated using CLUSTAL O (version 1.2.4) online tool.^27^ Exon boundaries in protein alignment data were obtained from the Ensembl database (release 100) using mouse genome assembly GRCm38.p6 and chick genome assembly GRCg6a.^28^

### 4.2 Ethical considerations, animal embryos, and embryo processing

Mouse experiments were approved by the Animal Review Board at the Court of Appeal of Northern Norrland (A34‐2019, A65‐2014, and A117‐2011) and performed in accordance to institutional guidelines and national laws. Under Swedish law, the use of chicken embryos at the gestational ages described here does not require ethical permission from the animal experimentation committee. *Barhl2* ^*GFP*^, *Math1* ^*LacZ*^, and *Math1* ^*nGFP*^ mice were maintained in a mixed genetic background and were genotyped as previously described.^4^, ^7^, ^29^ Chicken eggs were supplied by Stellan Hennström, Vännäs, Sweden. Staged mouse and chicken embryos were obtained and processed as previously described.^30^, ^31^, ^32^ In short, for immunohistochemistry embryos were fixed for 75 minutes in ice‐cold paraformaldehyde (4%), washed overnight in PBS and equilibrated and cryoprotected in sucrose (30% w/v in PBS) before embedding and freezing; for *in situ* hybridization, embryos were fixed at 4°C overnight in paraformaldehyde (4%) before washing and equilibrating in sucrose as above. At least three embryos for each group were analyzed as indicated in the figure legends.

### 4.3 Reverse transcription PCR for mouse and chick *Lhx9c* and *Lhx9ab* transcripts

Total RNA was extracted from embryonic mouse and chicken tissue using the RNeasy Mini Kit (Qiagen, #74104) and cDNA synthesized (AccuScript High Fidelity cDNA Synthesis kit, Agilent, #200820) according to manufacturer's instructions. PCR was performed using GoTaq Hot Start Green Master Mix (Promega, M512C) with primers designed to amplify *Lhx9c* and *Lhx9ab* cDNA from mouse and chick sequences (shown in Table 2).

**TABLE 2:** Oligonucleotide primers used in the study

Primer name	5′‐3′ sequence
Mouse Lhx9 canonical forward^a^	CGCACGAGCCAAATTCAGAAGG
Mouse Lhx9 canonical reverse^a^	CAATACTGTAAACTCATAGCCAAGTGG
Mouse Lhx9ab forward	GAACAAATCTTGGGGCATTACAGC
Mouse Lhx9ab reverse 1	CTTTACTTTACAGCTATGGTGCTCG
Chicken Lhx9ab forward	GAACAAATCATGGGGCATTACAGC
Chicken Lhx9ab reverse	GAAATGTACACTATATGTGACAGATGG

^a^ Mouse *Lhx9* canonical primers were also used for to amplify chicken *Lhx9* canonical cDNA.

### 4.4 Cloning of mouse and chicken *Lhx9c* specific probes and *Lhx9a* and *Lhx9c* expression plasmids

To create plasmids to use as templates for generation of *Lhx9c* specific riboprobes, first mouse and chicken cDNA was generated as described above. To isolate mouse and chicken *Lhx9c* specific cDNA fragments PCR was performed using GoTaq Hot Start Green Master Mix (Promega, M512C) and primers designed in this study (Table 2). The amplified PCR fragments were cloned into pGEM‐T Easy (Promega, A1360). To generate plasmids to express mouse *Lhx9a* or mouse *Lhx9c* with in COS‐7 cells, the coding region of Lhx9a or Lhx9c were cloned into the plasmid *pRP(exp*) vector which contains a separate *CMV‐GFP* expressing mini gene. The plasmids were constructed with a cloning service from VectorBuilder. The *Lhx9a* and *Lhx9c* expressing plasmids were assigned the following names: *pRP‐CAG‐Lhx9a:CMV‐GFP* and *pRP‐CAG‐Lhx9c:CMV‐GFP*.

### 4.5 *In situ* hybridization and probes

*In situ* hybridization was performed as described previously.^30^ The following probes were used: mouse *pan‐Lhx9* (full length *Lhx9* with a *Lhx9ab* 3′ end),^17^ mouse *Lhx9c* (this study), chicken *pan‐Lhx9* ^17^ and chicken *Lhx9c* (this study). Embryo samples were either wild‐type or carrying transgenes for *Math1* ^*LacZ*^, *Math1* ^*GFP*^, or *Barhl2* ^*GFP*^.

### 4.6 Antibody generation and validation

The antibody against Lhx9ab was raised in rabbit using the following peptide: EQILGHYSQTSRRLKIPC (Lhx9ab). The peptide was coupled to Keyhole limpet hemocyanin (Pierce) according to manufactures instructions and rabbits were inoculated with the antigen using standard inoculation procedures by Covance, USA. The specificity of the newly produced Lhx9ab antibody was assessed by examining recognition of overexpressed Lhx9ab protein in COS‐7 cells (Figure 6B). In short, COS‐7 cells were cultured and transfected, fixed, and analyzed as previously described using the following expression plasmids: Either GFP‐expressing plasmid (*CMV‐GFP; pEGFP‐N2* [Clontech, Mountain View, CA]) and empty vector (*pMT23*) or GFP‐expressing plasmid (*CMV‐GFP; pEGFP‐N2*) and *Lhx2*‐expressing plasmid,^21^ plasmid expressing both Lhx9a and GFP (*pRP‐CAG‐Lhx9a:CMV‐GFP*; this study) or a plasmid expressing Lhx9c and GFP (*pRP‐CAG‐Lhx9c:CMV‐GFP*; this study). Transfected cells were labeled by immunohistochemistry with GFP and Lhx9ab antibodies and counterstained with DAPI to lable the position of the nucleus. Each condition was performed in triplicate. In order to further examine the specificity of the Lhx9ab antibody, embryos from *Lhx2* ^*+/−*^ *; Lhx9* ^*+/−*^ *; Math1* ^*LacZ*^ crosses were immunolabeled with the newly produced Lhx9ab antibody as described below. This showed that while Lhx9ab was detected in control and *Lhx2* ^*−/−*^ embryos it was not detected in the spinal cord of *Lhx9* mutant embryos (Figure 7A). The antibody made in this study was generated by SIW in the laboratory of Professor Jane Dodd.

### 4.7 Fluorescent immunohistochemistry

Immunohistochemistry was performed on 20 μm cryosections as described previously^30^ using the following primary antibodies: guinea pig α‐pan‐Lhx9 (1:10 000),^21^ rabbit α‐LH2 (recognizes both Lhx2 and Lhx9, 1:1000),^6^ α‐Lhx9ab (1:10 000, generated in this study), goat α‐GATA4 (1:200, Santa Cruz, catalogue sc‐2537), chicken α‐GFP (1:3000, ABCAM, catalogue ab13970 RRID:AB_300798), chicken α‐GFP (1:1000, Aves Labs, catalogue GFP‐1020 RRID:AB_10000240). Secondary antibodies were as follows: donkey α‐rabbit—Cy3 (1:1000; catalogue 711‐165‐152, RRID: AB_2307443), donkey α‐guinea pig—FITC (1:500; catalogue 706‐095‐148, RRID:AB_2340453), donkey α‐goat Cy3 (1:500; catalogue 705‐165‐003) from Jackson ImmunoResearch Europe Ltd. and goat α‐chicken—FITC (1:1000; catalogue F‐1005, RRID:AB_2313516, Aves Labs, Oregon). DAPI was used to delineate nuclei. Primary antibodies were incubated overnight at 4°C, secondary antibodies were incubated at room temperature.

### 4.8 Microscopy and image processing

Samples were imaged using a Leica DM 6000B, DFC490, DFC360 FX, and Nikon Eclipse E800 microscopes. Images were processed for size, pixel density, and orientation using ImageJ. Merged images were produced by overlying single channel images in ImageJ. Figures were compiled in Adobe Creative Suite, 2019 or Affinity software 2020.

## CONFLICT OF INTEREST

The authors declare no conflicts of interest.

## AUTHOR CONTRIBUTIONS

**Benjamin Joel Wheaton:** Formal analysis (equal); investigation (equal); validation (equal); visualization (lead); writing – original draft (supporting); writing – review and editing (equal). **Sara Lea Häggström:** Formal analysis (equal); investigation (equal); resources (equal); validation (equal); visualization (supporting); writing – original draft (supporting); writing – review and editing (equal). **Mridula Muppavarapu:** Formal analysis (supporting); investigation (equal); resources (equal); validation (equal); visualization (supporting); writing – original draft (supporting); writing – review and editing (equal). **Luz María González‐Castrillón:** Formal analysis (supporting); investigation (supporting); validation (supporting); writing – review and editing (supporting). **Sara Ivy Wilson:** Conceptualization (lead); funding acquisition (lead); investigation (supporting); methodology (lead); project administration (lead); resources (equal); supervision (lead); writing – original draft (lead); writing – review and editing (equal).

## Data Availability

Data are available on request to the corresponding author.

## References

[1] Chen M , Manley JL . Mechanisms of alternative splicing regulation: insights from molecular and genomics approaches. Nat Rev Mol Cell Biol. 2009;10(11):741‐754. doi:10.1038/nrm2777 19773805 PMC2958924

[2] Wang X , He C , Hu X . LIM homeobox transcription factors, a novel subfamily which plays an important role in cancer (review). Oncol Rep. 2014;31(5):1975‐1985. doi:10.3892/or.2014.3112 24676471

[3] Helms AW , Johnson JE . Specification of dorsal spinal cord interneurons. Curr Opin Neurobiol. 2003;13(1):42‐49. 12593981 10.1016/s0959-4388(03)00010-2

[4] Wilson SI , Shafer B , Lee KJ , Dodd J . A molecular program for contralateral trajectory: Rig‐1 control by LIM homeodomain transcription factors. Neuron. 2008;59(3):413‐424. doi:10.1016/j.neuron.2008.07.020 18701067

[5] Pfaff SL , Mendelsohn M , Stewart CL , Edlund T , Jessell TM . Requirement for LIM homeobox gene Isl1 in motor neuron generation reveals a motor neuron‐dependent step in interneuron differentiation. Cell. 1996;84(2):309‐320. https://pubmed.ncbi.nlm.nih.gov/8565076/ 8565076 10.1016/s0092-8674(00)80985-x

[6] Liem KF Jr , Tremml G , Jessell TM . A role for the roof plate and its resident TGFbeta‐related proteins in neuronal patterning in the dorsal spinal cord. Cell. 1997;91(1):127‐138. 9335341 10.1016/s0092-8674(01)80015-5

[7] Helms AW , Johnson JE . Progenitors of dorsal commissural interneurons are defined by MATH1 expression. Development. 1998;125(5):919‐928. 9449674 10.1242/dev.125.5.919

[8] Molle B , Pere S , Failli V , Bach I , Retaux S . Lhx9 and lhx9alpha: differential biochemical properties and effects on neuronal differentiation. DNA Cell Biol. 2004;23(11):761‐718. https://pubmed.ncbi.nlm.nih.gov/15585134/ 15585134 10.1089/dna.2004.23.761

[9] Yang Y , Wilson MJ . Lhx9 gene expression during early limb development in mice requires the FGF signalling pathway. Gene Expr Patterns. 2015;19(1–2):45‐51. doi:10.1016/j.gep.2015.07.002 26220830

[10] Failli V , Rogard M , Mattei MG , Vernier P , Retaux S . Lhx9 and Lhx9alpha LIM‐homeodomain factors: genomic structure, expression patterns, chromosomal localization, and phylogenetic analysis. Genomics. 2000;64(3):307‐317. 10756098 10.1006/geno.2000.6123

[11] Kissinger CR , Liu BS , Martin‐Blanco E , Kornberg TB , Pabo CO . Crystal structure of an engrailed homeodomain‐DNA complex at 2.8 A resolution: a framework for understanding homeodomain‐DNA interactions. Cell. 1990;63(3):579‐590. 1977522 10.1016/0092-8674(90)90453-l

[12] Smagulova FO , Manuylov NL , Leach LL , Tevosian SG . GATA4/FOG2 transcriptional complex regulates Lhx9 gene expression in murine heart development. BMC Dev Biol. 2008;8:67. doi:10.1186/1471-213X-8-67 18577233 PMC2447832

[13] Birk OS , Casiano DE , Wassif CA , et al. The LIM homeobox gene Lhx9 is essential for mouse gonad formation. Nature. 2000;403(6772):909‐913. 10706291 10.1038/35002622

[14] Hu Q , Tian H , Meng Y , Xiao H . Characterization and tissue distribution of Lhx9 and Lhx9alpha in Chinese giant salamander Andrias davidianus. J Genet. 2016;95(3):683‐690. doi:10.1007/s12041-016-0685-3 27659340

[15] Bertuzzi S , Porter FD , Pitts A , et al. Characterization of Lhx9, a novel LIM/homeobox gene expressed by the pioneer neurons in the mouse cerebral cortex. Mech Dev. 1999;81(1–2):193‐198. 10330499 10.1016/s0925-4773(98)00233-0

[16] Tandon P , Wilczewski CM , Williams CE , Conlon FL . The Lhx9‐integrin pathway is essential for positioning of the proepicardial organ. Development. 2016;143(5):831‐840. doi:10.1242/dev.129551 26811386 PMC4813339

[17] Lee KJ , Mendelsohn M , Jessell TM . Neuronal patterning by BMPs: a requirement for GDF7 in the generation of a discrete class of commissural interneurons in the mouse spinal cord. Genes Dev. 1998;12(21):3394‐3407. 9808626 10.1101/gad.12.21.3394 PMC317230

[18] Retaux S , Rogard M , Bach I , Failli V , Besson MJ . Lhx9: a novel LIM‐homeodomain gene expressed in the developing forebrain. J Neurosci. 1999;19(2):783‐793. 9880598 10.1523/JNEUROSCI.19-02-00783.1999 PMC6782204

[19] Avraham O , Hadas Y , Vald L , et al. Transcriptional control of axonal guidance and sorting in dorsal interneurons by the Lim‐HD proteins Lhx9 and Lhx1. Neural Dev. 2009;4:21. doi:10.1186/1749-8104-4-21 19545367 PMC2704203

[20] Lee KJ , Jessell TM . The specification of dorsal cell fates in the vertebrate central nervous system. Annu Rev Neurosci. 1999;22:261‐294. 10202540 10.1146/annurev.neuro.22.1.261

[21] Wurmser M , Muppavarapu M , Tait CM , Laumonnerie C , Gonzalez‐Castrillon LM , Wilson SI . Robo2 receptor gates the anatomical divergence of neurons derived from a common precursor origin. Front Cell Dev Biol. 2021;9:668175. doi:10.3389/fcell.2021.668175 34249921 PMC8263054

[22] Tzchori I , Day TF , Carolan PJ , et al. LIM homeobox transcription factors integrate signaling events that control three‐dimensional limb patterning and growth. Development. 2009;136(8):1375‐1385. doi:10.1242/dev.026476 19304889 PMC2687467

[23] Sang M , Ma L , Sang M , Zhou X , Gao W , Geng C . LIM‐domain‐only proteins: multifunctional nuclear transcription coregulators that interacts with diverse proteins. Mol Biol Rep. 2014;41(2):1067‐1073. doi:10.1007/s11033-013-2952-1 24379077

[24] Gadd MS , Jacques DA , Nisevic I , et al. A structural basis for the regulation of the LIM‐homeodomain protein islet 1 (Isl1) by intra‐ and intermolecular interactions. J Biol Chem. 2013;288(30):21924‐21935. doi:10.1074/jbc.M113.478586 23750000 PMC3724647

[25] Grigoriou M , Tucker AS , Sharpe PT , Pachnis V . Expression and regulation of Lhx6 and Lhx7, a novel subfamily of LIM homeodomain encoding genes, suggests a role in mammalian head development. Development. 1998;125(11):2063‐2074. 9570771 10.1242/dev.125.11.2063

[26] Coordinators NR . Database resources of the National Center for biotechnology information. Nucleic Acids Res. 2018;46(D1):D8‐D13. doi:10.1093/nar/gkx1095 29140470 PMC5753372

[27] Sievers F , Wilm A , Dineen D , et al. Fast, scalable generation of high‐quality protein multiple sequence alignments using Clustal Omega. Mol Syst Biol. 2011;7:539. doi:10.1038/msb.2011.75 21988835 PMC3261699

[28] Yates AD , Achuthan P , Akanni W , et al. Ensembl 2020. Nucleic Acids Res. 2020;48(D1):D682‐D688. doi:10.1093/nar/gkz966 31691826 PMC7145704

[29] Lumpkin EA , Collisson T , Parab P , et al. Math1‐driven GFP expression in the developing nervous system of transgenic mice. Gene Expr Patterns. 2003;3(4):389‐395. 12915300 10.1016/s1567-133x(03)00089-9

[30] Kropp M , Wilson SI . The expression profile of the tumor suppressor gene Lzts1 suggests a role in neuronal development. Dev Dyn. 2012;241(5):984‐994. doi:10.1002/dvdy.23777 22419569

[31] Laumonnerie C , Tong YG , Alstermark H , Wilson SI . Commissural axonal corridors instruct neuronal migration in the mouse spinal cord. Nat Commun. 2015;6:7028. doi:10.1038/ncomms8028 25960414

[32] Hamburger V , Hamilton HL . A series of normal stages in the development of the chick embryo. J Morph. 1951;88:49‐92. 24539719

